# Selected Aspects Related to Medicinal and Aromatic Plants as Alternative Sources of Bioactive Compounds

**DOI:** 10.3390/ijms22041521

**Published:** 2021-02-03

**Authors:** Radu Claudiu Fierascu, Irina Fierascu, Anda Maria Baroi, Alina Ortan

**Affiliations:** 1 National Institute for Research & Development in Chemistry and Petrochemistry—ICECHIM, 060021 Bucharest, Romania; fierascu.radu@icechim.ro (R.C.F.); baroi_anda@yahoo.com (A.M.B.); 2 Department of Science and Engineering of Oxide Materials and Nanomaterials, University “Politehnica” of Bucharest, 011061 Bucharest, Romania; 3 Veterinary Medicine of Bucharest, University of Agronomic Sciences, 011464 Bucharest, Romania; alina_ortan@hotmail.com

**Keywords:** medicinal plants, bioactive compounds, biomedical applications, industrial applications, nanotechnology

## Abstract

Natural compounds obtained from different medicinal and aromatic plants have gained respect as alternative treatments to synthetic drugs, as well as raw materials for different applications (cosmetic, food and feed industries, environment protection, and many others). Based on a literature survey on dedicated databases, the aim of the present work is to be a critical discussion of aspects regarding classical extraction versus modern extraction techniques; possibilities to scale up (advantages and disadvantages of different extraction methods usually applied and the influence of extraction parameters); and different medicinal and aromatic plants’ different applications (medical and industrial applications, as well as the potential use in nanotechnology). As nowadays, research studies are directed toward the development of modern, innovative applications of the medicinal and aromatic plants, aspects regarding future perspectives are also discussed.

## 1. Introduction

**Figure 1:**
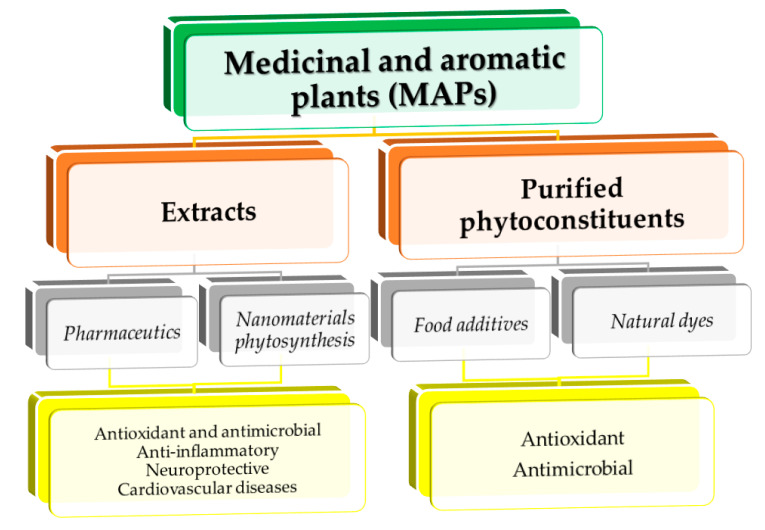
Some of the potential applications of medicinal and aromatic plants.

Natural compounds obtained from different medicinal and aromatic plants (MAPs) have gained respect as alternative treatments, as well as raw material for different applications. Medicinal plants are a source of bioactive compounds that act as drugs in traditional treatments [1]; meanwhile, aromatic plants represent a rich source of essential oils, which can be used for their aroma and flavor [2]. MAPs are also used in cosmetics, functional food, or natural dyes production [3], thousands of species are all over the world being explored and exploited [4]. Another recent application is in the nanotechnology area, where the plant extracts’ phytoconstituents act as reducing and capping agents for the reduction of metallic ions from solutions in order to obtain different metallic nanoparticles, with further biomedical or industrial applications [5] (Figure 1).

The increased population led to a higher utilization of these plants, so their residues are proportional, with a huge amount of biomass generated as by-products [6], representing a growing market in the natural-based products [7]. The general use of MAPs all over the world is not homogenic, due to different factors: (i) in developed countries, even if the demand for natural treatments is high, profits of the growers and producers remain low because of the existing intermediaries which increase the price, as well as the lack of organization and networking by the poor collectors of medicinal plants from the wild; (ii) rigorous regulations and documentations requirements; and (iii) in less developed countries, there are poor traceability mechanisms from plant to population [8].

In this context, there are many studies reporting biological effects that can be attributed to MAPs or derived products, against several diseases, such as cancer, neurological, respiratory, inflammatory, cardiovascular diseases, and many others [9,10,11,12]. Using phytoconstituents or natural-based products as complementary treatments improved the oxidative status, which is due to their activity in compensating the inefficacy of the endogenous defense systems and in the enhancement of the overall antioxidant response [13,14]. Under stress conditions, the human body produces more reactive oxygen and nitrogen species (ROS/RNS) than enzymatic and non-enzymatic antioxidants, which is conducive to cell damage and health problems [15], and biological active products have a crucial role in combating oxidative stress.

In addition to traditional medical applications of medicinal and aromatic plants, there is the possibility of using them in cosmetic products, feed or food additives and preservatives, or as a viable tool for biotechnological applications, such as the enhancement of secondary metabolites by genetic engineering [16,17,18].

Based on a literature survey on dedicated databases (Scopus, ScienceDirect, SpringerLink, PubMed, Web of Science), the aim of this review paper is to be a critical discussion of aspects regarding classical extraction versus modern extraction techniques; possibilities to scale up (advantages and disadvantages of different methods usually used and the influence of extraction parameters); and different medicinal and aromatic plants’ different applications (medical and industrial applications, as well as the potential use in nanotechnology). In addition, future perspectives are critically discussed.

## 2. Classical Extraction Versus Modern Extraction Techniques: Possibilities to Scale up

Classical methods were developed during the time, such as a simple approach involving only water, herbs, and energy [19]. During the optimization process, the economic costs and energy necessary for obtaining different bioactive compounds tends to decrease, leading to higher yields of target compounds. Instead of maceration, decoction, or infusion, in the past, researchers developed methods such as Soxhlet, Clevenger, Kumagawa, or Likens–Nickerson simultaneous distillation–extraction, which, in turn, are replaced in the present day with modern equipment such as microwave-assisted extraction, supercritical fluid extraction [20], or ultrasound-assisted extraction [21]. Some examples are presented in Table 1 in order to present conditions for classical extraction versus necessary conditions for modern methods.

From the data presented in Table 1, it can be seen that for the same plant, extraction conditions may vary, depending on each method: for classical methods, the extraction time and the amount of solvent used are higher than for modern techniques compared to the obtained extraction yield (i.e., *Satureja hortensis* L. or *Hippophae rhamnoides* L.), thus leading to new approaches and perspectives. Recently, researchers proposed the introduction of a “*Green Certificate*”, which is based on weighted penalty points and the use of a color code in which reagent toxicity and volume, energy consumption, and the quantity of generated wastes (in the extraction step) are the main parameters used for the quantification of the “green” character [39,40,41]. All of these parameters contribute to a “greener character of the technique”, which is sometimes more important than the easiness of obtaining results. The classical approaches usually provide the lowest green certificate values, which is due to the energy and high amounts of solvents consumption. The “green certificate” is based on the application of a color code associated to a letter, class A being the “greenest” one [41]. The selection of a suitable solvent is crucial to improve the extraction yields, and moreover, the amount of resulted waste after the extraction. Polar solvents are commonly used, such as ethanol, methanol, and isopropanol [42], but the trends in “*green chemistry*” are going toward novel solvents with less toxicity as natural deep eutectic solvents (NADES) [43]. Espino and coworkers developed and optimized ultrasound-mediated extraction for phenolic compounds from *Larrea cuneifolia* Cav. 1800, which is a medicinal plant from the *Larrea* genus used in the Argentinian folk medicine [44]. They chemometrically optimized the extraction conditions, obtaining better results than using classical solvents in terms of resulted wastes: thus, for conventional techniques (maceration, decoction, heat reflux), wastes are in the range 1.5–2.85 penalty points for waste calculated according to the methodology previously mentioned [39,40,41], while for modern techniques, such as microwave and ultrasound-assisted extractions, wastes are in the range of 1–1.5 penalty points for waste, which are calculated according to the same protocol. These values of penalty points of the wastes offer a green certificate of class A (with a value in the range 100–90) and class B (89–80). Considering the results presented by the authors, the use of NADES and modern extraction techniques (such as ultrasound extraction) could be successfully applied for the scale-up of the procedure.

Another “*green solvent*” is carbon dioxide, which can be used as the main solvent for supercritical fluid extraction, having advantages such as non-toxicity and thermodynamic parameters, which facilitates its use in the supercritical state [45], being a suitable solvent first for non-polar molecules (lipids, terpenes, etc.), followed by more polar molecules [46]. In the case of *Salvia officinalis* L., Jokić and coworkers optimized a supercritical CO_2_ extraction method for terpenes and phenolic compounds, obtaining increased yields of recovery (7.4%) by varying the utilized pressure (15 or 20 MPa) [33] in only 90 min of extraction; meanwhile, Miguel and collaborators, through hydro-distillation obtained in 180 min a decreased yield of recovery (2%) [34]. Moreover, the quality of the obtained compounds can be modified depending on the extraction method used; Ollanketo and coworkers demonstrated that pressurized hot water extraction is a highly promising alternative to conventional solid–liquid techniques, in terms of final application of the recovered compounds; the highest antioxidant activities did not correspond to the maximum recovery yields, but the antioxidant activity was highest when pressurized hot water (PHW) was used as the extracting solvent instead of the maceration method [35]. In this case, the quality of the compounds is influenced by the extraction conditions, in which an increased time and temperature lead to a decrease of biological effects of the obtained compounds. In addition, some compounds do not respond to a classical or modern solvent (such as NADES) extraction, and it is necessary to optimize an enzymatic process and break hydrogen or hydrophobic bonding, which keeps them trapped in the polysaccharide–lignin network [47].

As a pro argument for modern extraction techniques, in addition to the reduced costs and energy, there is the use of a cascade of different solvents, which is due to the existence of a complex matrix of MAPs; this approach is conducive to the recovery of a large range of bioactive compounds. This is the case of Algerian *Thymus munbyanus* Boiss. & Reut., 1852, where acetone, ethanol, and water were used in successive pressurized extractions [38]. The recuperation of the solvents, especially the toxic ones such as acetone, can be performed under vacuum conditions, removing the possibility of contamination on the environment or on the final products. In addition to oxygenated monoterpenoids, such as camphor (11.7%) and geraniol (7.5%), and sesquiterpenoids and monoterpenoids, such as (E)-nerolidol (13.7%), terpinen-4-ol (10.6%), and camphor (7.6%), researchers also obtained geranyl acetate (6.3%) and β-terpinyl acetate (5.1%), caryophyllene oxide (5.1%) and borneol (5.6%), respectively β-terpinyl acetate (4.8%) and linalool (4%).

A proper balance in choosing the methods and setting operational parameters could lead to an optimized process, obtaining different compounds from a plant matrix, which in other conditions would be damaged or could not be obtained [48]. The structure of target molecules can influence their solubility at different conditions of high pressure; in extreme conditions, interactions and aggregations or even their re-adsorption are possible. Using moderate conditions such as medium pressures or temperatures automatically can result in lower costs and energies, which is beneficial for all the production and processing chain. Moreover, for modern extraction methods, for which it is not necessary to use organic or toxic solvents, the obtained bioactive compounds could achieve, in certain conditions, a “green characteristic” and can be used in further applications such as the design of foods with improved functionality or medical care.

Modern extraction methods have attracted a great amount of interest in the last few years, especially due to their scale-up possibilities and their ability to provide superior quality extracts with economic benefits; a scientific and analytical approach must be conducted for this step. The classical extraction method can be also scaled up, but factors such as instrumentation, batch/flow process, kinetics, economics, energy consumption, and amounts of wastes tilt the balance toward the scale up of non-conventional extraction techniques, such as microwaves or ultrasonic-assisted, supercritical fluid, negative cavitation, pressurized fluid, etc. [49]. In some cases, the adequate parameters for lab scale can be used in scale-up process [50], but in other cases, maintaining the same conditions can led to a decreased recovery yield when the scaled-up method is applied [51]. In the optimization process, there must be a balance between the parameters, and according to Belwal and coworkers, every method must prove its maturity level through technology readiness levels (TRL) [49].

## 3. The Influence of Extraction Conditions

For each method used as an extraction technique, optimization of the parameters is mandatory. The reaction parameters that can influence the extraction process are the used solvent, temperature, ratio of vegetal material/amount of solvent, pH, extraction time, and factors related to the raw material matrix [52].

**Figure 2:**
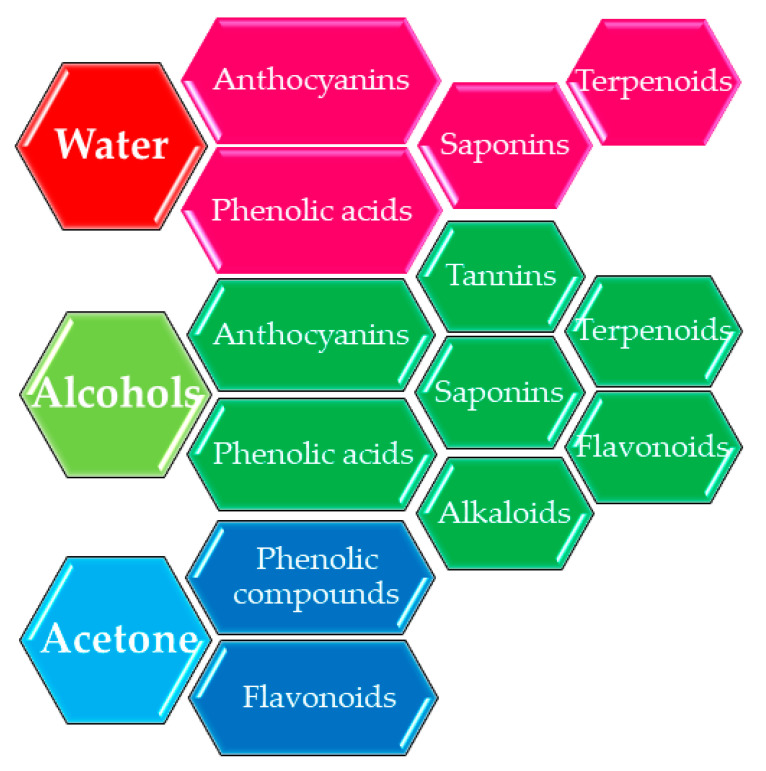
Commonly used solvents in the extraction process.

According to the experiments performed by different authors, the most suitable solvents for bioactive compounds extraction are water for anthocyanins, phenolic acid, saponins, terpenoids recovery [53,54,55,56], and alcohols such as methanol or ethanol, alone or as mixtures, for anthocyanins, phenolic acids, flavonoids, tannins, saponins, or terpenoids recovery [57,58]. Alcohols have the property of increasing cell permeability by affecting the phospholipid bilayer of the membrane, permitting a good transfer of bioactive compounds into the solvent. In addition, the water/alcohol mixtures permit a good recovery yield, especially for phenolic compounds, which have a good solubility, due to the alcohol presence (Figure 2).

In the last years, the application of neoteric solvents received special interest, in order to minimize the use of toxic components and maximize the extraction efficiencies, with an emphasis on reducing their toxicity after usage [59]. This category includes ionic liquids and deep (natural) eutectic solvents, which were used due to their possibility toward tailored-extraction, thus increasing extraction efficiency for complex matrixes. Ionic liquids are suitable for different bioactive compounds, such as alkaloids [60] or phenolic compounds [61] due to the good miscibility of target compounds with the solvent. Eutectic solvents have the same properties with ionic liquids, and beside this, they are less toxic and more biodegradable [62]. In addition, bio-based solvents received special interest, such as 2-methyltetrahydrofuran, limonene, or 2-methyltetrahydrofuran, successfully replacing solvents such as n-hexane and toluene with lower production costs and increased biodegradability [63]. Some of these solvents are not commercially available, and they are not still used at an industrial level. Nowadays, the experiment must be carried out with respect to the balance between environment and production costs, in order to achieve full sustainability in the process implementation.

Depending on the plant matrix, temperature is another important parameter that is necessary in the optimization process. Increased temperature can lead to a higher solubility of the analyte, but at the same time, it can lead to the degradation of thermo-sensitive compounds [64]. In the processes where neoteric solvents are used, the temperature significantly modifies the properties of the solvent, thus modifying the solubility of the analytes [65].

The role and optimization of time, pH, solid–liquid ratio, or pressure are discussed in different papers [66,67,68,69,70], as these are significant parameters that can influence the extraction efficiency. Moreover, it cannot be stated than one parameter is more important than another, each of them having a tremendous influence on the overall extraction process results.

## 4. Different Medicinal and Aromatic Plants—Different Applications

### 4.1. Medical Applications

Due to the fact that this review paper intends to be a critical discussion on different aspects related to the applications of medicinal and aromatic plants even though they are used as such, or as extracts, nanomaterials, or purified bioactive compounds, it is impossible to approach this topic for all the existing plants. We will focus on a few examples of medicinal and aromatic plants, with a wide spread and knowledge, based on the newest reports, in order to demonstrate the importance of these plants for treating/preventing diseases of the century (cardiovascular or neurodegenerative diseases, diabetes, etc.) or even for simple biological effects (antibacterial, antioxidant).

#### 4.1.1. *Origanum* spp.

Oregano belongs to the Lamiaceae family, and within the genus Origanum, there are three groups, 10 sections, 38 species, 6 subspecies, and 17 hybrids [71]. Among the different Origanum species, several are commonly used for culinary purposes (especially as spices), such as the Greek oregano (*Origanum vulgare* L. ssp. *hirtum* (Link) Ietswaart), Marjoram (*Origanum marjorana* L.), Turkish oregano (*Origanum onites* L.), round-leaved oregano (*Origanum rotundifolium* L.), or Syrian oregano (*Origanum syriacum* L.) [72,73,74]. The *Origanum* spp. plants, especially leaves, are rich in terpenes (monocyclic terpenes—from which the most concentrated are carvacrol and thymol; bicyclic monoterpenes such as thujene, sabinene, camphene, α and β pinene; acyclic monoterpenes such as linalool; sesquiterpenes such as β-bisabolene; triterpenoids such as ursolic and oleanolic acids) [75] and phenolic compounds (hydroquinone and derived compounds; phenolic acids: p-hydroxybenzoic, vanillic, syringic; flavonoids) [72], but the composition depends on different factors (species, cultivation area, environment conditions, harvesting time, phenopase, etc.).

Even if it is used as such or encapsulated in different matrixes, the oregano essential oil (OEO) has predominant medical applications. In the last few years, researchers reported in vitro antibacterial, antioxidant, and anti-inflammatory activities for the essential oil [76,77,78,79], in the year 2020, Khan and co-workers reported the results of an in vivo study regarding a wound healing based on capsules of co-polymer of poly (L-lactide-co-caprolactone) (PLCL)/silk fibroin loaded with different concentrations of OEO [80]. The activity of oregano essential oil can balance the production of ROS, playing a positive role in the healing process. In addition, it has the property of boosting the granulation, re-epithelialization, better organization of collagen fibers, as well as capillary network formation. Avola and coworkers described biological effects of OEO in the restoring of physiological cell homeostasis, using human keratinocytes NCTC 2544 treated with interferon-gamma (IFN-γ) and histamine (H) [81]. OEO diminished the release of a variety of pro-inflammatory mediators such as iNOS (inducible nitric oxide synthase), ICAM-1 (inter-cellular adhesion molecule 1), and COX-2 –(cyclooxygenase-2), reducing ROS development, 8-OHdG (8-hydroxy-2′-deoxyguanosine) formation, and maintaining the protein proliferating cell nuclear antigen (PCNA) expression without influencing cell viability. The OEO was proven to play an important role in preserving the extracellular matrix components, remodeling, and tissue healing. For re-epithelialization of the skin after its injury, OEO has the property of improving NCTC 2544 cell proliferation. In this study, the beneficial effects were attributed to carvacrol, which is the main constituent in OEO.

OEO has also a beneficial effect when it is encapsulated in polymeric matrixes, and different studies being conducted toward both developing new materials that can limit the volatility of the essential oil and obtaining a controlled release for the topical application. This is the case of nanocomposite films based on poly(vinyl alcohol) (PVA) and alphachitin nanocrystals (α-CHNC, from shrimp and lobster), which are conductive to a “green” character of the treatment [82]. In general, one of the main drawbacks of the application of essential oils in wound dressing is related to their very low solubility or absent solubility in aqueous solutions; cross-linking and/or other additives are usually necessary [82].

One of the major health problems in the world is the increased mortality for metabolic diseases and diabetes mellitus [83]. OEO is a solution for antihyperglycemic activity, being a good candidate to prevent and/or treat diseases arising from oxidative stress, such as diabetes mellitus, by the inhibition of α-amylase and α-glucosidase [84]. For this effect, the responsible phytoconstituent is 4-terpineol, which was found to be the most abundant compound in OEO; the recorded inhibition of α-amylase activity was 81.4%, while the inhibition of α-glucosidase activity was 50.5%, which is within the results range obtained in other studies [77].

OEO is known as one of the best candidates to be used as preservatives against the *Bacillus* species (associated with food spoilage), which is a property attributed to the presence of oxygenated terpenes (terpenoids) [85] or in association with other treatments, such as gamma irradiation, as well as against different pathogens (*Escherichia coli*, *Salmonella typhimurium,* and *Listeria monocytogenes*) [86]. Moreover, some review papers present recent developments regarding the use of OEO and nanomaterials based on oregano extracts in medical care or other applications, such as the food industry [87,88,89]. OEO can reduce the water vapor permeability (WVP) and enhance the contact angle, transparency, and moisture value of final films containing it, which is necessary for the development of different coatings.

#### 4.1.2. *Thymus* spp.

*Thymus genus*, belonging to the Lamiaceae family, consists of 250–350 taxa widespread all over the world. Among these species, *Thymus vulgaris* L. 1753 is the most commonly found, and it is used as a culinary product or as medicinal remedy. The plant is rich in essential oil (the most abundant in flowers), which has as its main chemical classes terpenes, terpene alcohols, phenolic derivatives, ketones, aldehydes, ethers, and esters [90]. Depending on the species, the chemical composition can vary: monoterpene phenols, with the isomers thymol and carvacrol, are usually two major compounds in most species (*T. vulgaris*, *T. capitatus*, *T. pulegioides*, *T. pubescence*, *T. daenensis*, *T. transcaspicus*, *T. serpyllum*, *T. fallax*, *T. kotschyanus*, *T. reolutus* Celak) [91], while *T. caespititius*, *T. camphoratus*, and *T. mastichina* are rich in α-terpineol, linalool, and 1,8 cineole [92]; *T. algeriensis* has α-pinen as its main constituent [93], and geraniol is specific for the Romanian native *T. glabrescens* [94].

In order to eradicate pathogenic yeasts strains, such as *Candida albicans*, *C. krusei*, or *C. glabrata*, Muslim and Hussin proposed a recipe based on the synergistic effects of *Thymus kotschanus* Boiss & Hohen essential oil and ketoconazole for decreasing the incidence of candidiasis among vulnerable individuals with underlying diseases (antibiotic therapy, AIDS, etc.) [95]. In this case, the role of the essential oil was to inhibit the microbial population and to increase the permeability of fungal membranes, making them more sensitive to other antifungal agents, such as synthetic drugs. As a perspective of using EO for downexpressing the Als (Agglutinin-like sequence), a biofilm-associated gene is the development of future in vivo studies to optimize the effects of temperature, pH, host responses, as well as drug resistance to these EOs.

In 2020, Najafloo and coworkers published a review paper that presented the applications in the area of antibacterial wound dressing of thymol-incorporated materials, based on essential oil’s therapeutic properties: antioxidant, anti-inflammatory, local anesthetic, antinociceptive, cicatrizing, antiseptic, and particularly antibacterial and antifungal [96]. These delivery systems can be formed by lipid-based nanocarriers, polymer-based nanoparticles, fiber capsules, or hydrogels, all of them having application in wound treatment, the active bio-compound thymol, having the property to modulate the release production of reactive species, including nitric oxide, TNF-α, and IL-1β cytokines and growth factors such as TGF-1β, thus stimulating re-epithelialization, angiogenesis, and the formation of granulation tissue.

In addition, terpenes extracted from the aerial parts of *Thymus* spp. are potent cardioprotective agents, protecting against biochemical and histopathological changes in the heart tissue of animals by restoring the activities of endogenous antioxidant enzymes [97]. At heart diseases, there is an increased generation of reactive oxygen species such as superoxide anion (O_2_^−^) and hydroxyl radicals (OH^−^), and thymol has the ability to remove the damage of ROS, increasing the endogenous antioxidant enzyme activities such as superoxide dismutase (SOD), catalase (CAT), glutathione peroxidase (GPx), or glutathione-s-transferase (GST). Other applications to be further developed using the main constituents of *Thymus* spp. in different forms can be beneficial in biomaterials development and tissue regeneration; recent studies [98,99] present the antiviral potential of medicinal and non-medicinal plants applied for the contemporary pandemic agent COVID-19; considering the proven biomedical potential of *Thymus* spp., their application in this area should definitely be explored.

#### 4.1.3. *Salvia* spp.

*Salvia* is the largest genus of the Lamiaceae family, containing over 900 species, and beside the therapeutical properties that confer the possibility of being used in medical applications, it can be used as a flavoring agent, perfume additive, and condiment [100]. All parts of the plant are rich in antioxidant and antimicrobial phytoconstituents, such as essential oils, terpenes, flavonoids, phenolic acid, and steroids. In addition to leaves and flowers, which can be used from most of the plants, the roots have beneficial properties, and moreover, the roots of *Salvia miltiorrhiza* Bunge are a good instrument for biotechnological applications, such as the enhancement of secondary metabolites by genetic engineering and elicitor treatment [101].

The anxiolytic effect of a *Salvia* extract was studied by Lin and coworkers, using the elevated plus-maze test (EPM) and the hole-board test (HBT) [102]. In the cited study, the application of *Salvia* extract on mice showed a significant increase in their head-dip counts and duration compared to the control group (diazepam and flumazenil), recommending this plant as a potential anti-anxiety drug.

In addition, the combination of *Salvia miltiorrhiza* Bunge. and *Carthamus tinctorius* L. extracts acts synergistically, producing a combined effect, enhanced compared with the individual effects in targeting complex diseases such as diabetes mellitus, hypertension, and related cardiovascular diseases [103]. Obesity, hypertension, and diabetes share a common pathological relationship with metabolic syndrome and from one chronic state, it can be transformed to another. The mixture of the compounds has the ability to inhibit the formation of acellular capillaries (which can be related to the development of vascular aneurysms), as well as regulator effect on glucokinase induction, AMPK-α/phosphorylated AMPK-α, insulin receptor substrate-1, and PPARγ, thus mediating the glucose metabolism.

As a future perspective, the pharmacokinetics and pharmacodynamics of the natural compounds recipe require further preclinical and clinical investigation in order to be applied at a large industrial scale as natural drugs.

### 4.2. Industrial Applications

In addition to medical applications, bioactive compounds from medicinal and aromatic plants proved to have important properties, which made them good candidates for industrial applications. Due to their antioxidant and antimicrobial effects, these natural products can be used especially in the food, pharmaceutical, and perfume industries (Table 2).

Even from the beginning of the 1990s, the interest in using natural compounds in industrial applications started in controlling the postharvest disease of citrus fruits [104] and continued until nowadays, using edible coatings (edible chitosan coatings incorporated with *Thymus capitatus* essential oil) as an alternative method to prolong the shelf life of perishable fruits (strawberries) [105]. The treatment can be applied to postharvest fruits to keep them in good conditions for more than 15 days, and their antioxidant properties are not affected. Jemaa and coworkers applied as a food preservative the essential oil of *Thymus capitatus*, the antimicrobial activity being attributed to carvacrol [106]. *Eryngium campestre* essential oil encapsulated in chitosan nanoparticles was developed to reduce the microbial counts and to extend the shelf life of sweet cherries, farnesene being identified as the main responsible compounds for the antimicrobial effects [107]. For the cases of encapsulation, different physicochemical characterizations are needed in order to optimize these materials for specific applications.

In the food industry, natural compounds can be used as natural packaging materials, which have the ability to increase the shelf life of meat products. Encapsulated in chitosan particles, these compounds can protect against different bacteria (*Pseudomonas* spp., *Listeria monocytogenes,* etc.), and moreover, they have the property to inhibit lipid oxidation [108]. Poly lactic acid/nanochitosan composite film enriched with *Polylophium involucratum* was developed for prolonging the shelf life of chicken fillets during refrigerated storage for 10 days [109]. These packaging films used for removing adverse sensorial properties due to microbial attack are environmentally friendly materials that are beneficial for human health, replacing petroleum-based plastic packaging. Rehman et al. presented different plants such as *Syzygium aromaticum*, *Mentha piperita*, *Salvia rosmarinus*, or *Eucalyptus globulus* as potential natural sources for food packaging [110].

In addition to these applications, there are edible coatings, which are considered as a part of the final product, and they are used to increase the oxidative stability during the storage of different foods; this approach is considered a preservation technique. Hosseini and coworkers developed a recipe based on whey protein concentrate, carboxymethyl cellulous, glycerol, and rosemary extract to improve the color and oxidative properties of the stored sunflower seeds [111]. By adding carboxymethyl cellulose, the synergistic effect with the extract prevents the formation of hydroperoxides and conjugated dienes [112], and in the case of sunflower seeds, it provides the highest tensile strength, coating percentage, and elongation of the film [111].

With a special respect to the natural products recipe, a great development started in the last few years for natural insecticidal recipes, which are less harmful to humans and the environment. *Th. alternans* and *T. montanum* subsp. *Jailae* were studied by Pavela and coworkers as insecticide against *Musca domestica* L., *Culex quinquefasciatus* Say, and *Spodoptera littoralis* (Boisd.) [113]. The insecticidal effect can be attributed to the synergistic effect of the individual compounds found in these species, and further studies are needed in order to elucidate the mechanism of action.

In addition, MAPs, such as *Origanum vulgare* subsp. Hirtum, *Foeniculum vulgare* Mill, *Pimpinella anisum* L., and their extracts can be used as a novel feed additive in turkey production, having beneficial effects, such as food microbial safety or even can enhance production performance [114] as “green pesticides” to limit the use of hazardous synthetic pesticides (the case of *Heracleum persicum* and *Achillea millefolium* essential oil against *Plodia interpunctella*, citronellal from *Cymbopogon winterianus* against *Spodoptera frugiperda* larvae, or rosemary oil applied against *Agriostes obscurus* larvae) [115] or in the zootechnological field to enhance animals’ performance and health [116]. Moreover, purified bioactive compounds from MAPs (such as *Calendula officinalis* L., *Lavandula vera* DC, *Artemisia absinthium* L.) or even plant extracts (as a whole) have potential applications in the cosmetic industry, which nowadays is focused on new technologies and explores alternative sources of raw materials; the trends in this direction are based on the use of plant-origin components having polyfunctional properties and long-lasting effects [117]. In this domain, there is ongoing in-depth research for identifying natural resources for sunscreen cosmetics; bioactive compounds such as green tea polyphenols, *Rosa damascene* flower extracts, aromatic compounds isolated from lichens, flowering tops of *Dracocephalum moldavica* and *Viola tricolor*, and aromatic and flavonoid compounds from saffron, *Crocus sativus,* were evaluated for their effects [118]. In addition, compounds such as a-pinene, b-pinene, limonene, cymene, linalool, cis-2-methoxycinnamic acid, and cinnamaldehyde are used for their anti-melanogenic and anti-aging properties [119,120].

Instead of synthetic additives, which are toxic and harmful to humans and the environment, the interest regarding the application of natural extracts from plants in this area has increased. These substances can be successfully applied as additives in steel production for developing corrosion-resistant materials [121]. The beneficial effects of the newly developed coatings can be increased by decreasing the cost of production, natural compounds being a cheap raw material, but the disadvantage of their use is over the long term, being degradable under specific conditions (UV light, the possibility of oxidizing or evaporating) [122]. Coatings based on bioactive compounds from MAPs can also have antimicrobial [123,124] or antifouling applications [125].

Natural products can be used as a natural resource of photostabilizers in coatings, instead of hindered amine light stabilizers or inorganic nanoparticles, which have the disadvantage of leaching. Tannin from *Pinus brutia* Ten. has improved the color stability and surface quality of the coated wood during artificial weathering [126] and coatings based on Chinese fir bark extract enhanced the weathering resistance of wood [127].

### 4.3. Applications in Nanotechnology

In the last decades, nanotechnology has offered a series of valuable tools for improving our daily life. Within this area, the application of different plants (including MAPs) led to the development of a new research field, nanoparticles phytosynthesis. As a general rule, the plant’s phytoconstituents, acting as both reducing and capping agents, determine the final morphology and size of the obtained nanoparticles as well as contribute to an increase in biological activity and a reduction of potential toxicity. Our group previously reviewed the most recent findings in the application of phytosynthesized NPs as antimicrobial and antitumoral agents, as well as the results regarding their toxicological potential [133], plants such as *Azadirachta indica* A. Juss., *Berberis vulgaris* L., *Coriandrum sativum* L., *Mentha pulegium* L., *Myrtus communis* L., *Salvia hispanica* L., or *Tribulus terrestris* L. (just to name few examples) offer proper extracts to obtain silver, gold, or ZnO nanoparticles. Moreover, these nanoparticles, in particular those phytosynthesized with the help of medicinal plants such as *Artemisia vulgaris* L., can find application in other medical applications, such as antiviral agents for chikungunya virus [134], antiparasitic activity against yellow fever mosquito (dengue fever vector) [135], or against different infections [136].

The subject of phytosynthesized nanoparticles is very vast and itself is a main topic for a review paper. For this manuscript, we focused on the discussion of some important aspects related of the general mechanism of action and applications in some domains related to those ones in which “bioactive compounds from MAPs” can be used.

As previously stated, the plant extracts constituents act as reducing agents for metallic salts, also having the role as stabilizing/capping agents and inducing the polymorphism of the obtained nanoparticles. Moreover, the effect of nanoparticles is enhanced by the synergistic effect of bioactive compounds, especially in antimicrobial and antioxidant applications [137].

The mechanism of cellular action of these nanoparticles is similar for several types of application: the nanoparticles have the ability to inhibit the cellular mechanism by DNA damage, which is conducive to the death of the target cell. In addition, the formation of reactive oxygen species, including hydrogen peroxide, leads to oxidative stress and subsequent cell damage [138]. The phytosynthesized nanoparticles decrease the glycated hemoglobin antioxidant defense, presenting and reducing the metalloproteinases activity that is conducive to an anti-inflammatory effect [139]. Functionalizing nanoparticles with natural antioxidants increases their stability and biocompatibility [140].

In addition to medical applications (antimicrobial, antioxidant, antitumoral, etc.), “green nanoparticles” are used for environmental applications and for waste water depollution as “green catalysts” [141,142].

## 5. Conclusions and Future Perspectives

The use of medicinal and aromatic plants has strong roots in the traditional medicine since antiquity, from the treatment of minor illnesses to more contemporary diseases [143]. Since then, evolving from simple maceration and decoction, based on modern approaches, research has reached a top level of technologicalization in which bioactive compounds are successfully recovered or even purified [144]. Our group presented in a published paper the most recent findings and approaches for the recovery of phytoconstituents from MAPs [1], so now, we will focus for this paper on future perspectives of using these compounds for further applications.

One of the most spectacular uses of MAPs is in the biotechnological domain, where omics technologies have a crucial role. For an increased production of natural bioactive compounds, the elucidation of the biosynthetic pathway will help directly and efficiently obtain active compounds of different plants in hairy roots systems. In addition, a good knowledge and usage of biotic and abiotic elicitors can dramatically improve the production of targeted active compounds. If some studies proposed an exact methodology and models for the biosynthesis and regulation of active compounds (for example, in *Salvia miltiorrhiza* Bunge., as previously presented), further studies are needed for other medicinal plants and for similar developments.

The road “from plant to pharmacy shelf” is long, sinuous, and hardly achievable, but with tremendous rewards at the end: the development of commercial products that could increase the life quality and treat a series of illnesses and conditions with high social and economic impact on the society as a whole.

Knowing the beneficial effect of metallic nanoparticles in the medical applications and following the model of chemical synthesized Fe_3_O_4_ magnetic nanoparticles that have effects in neurodegenerative diseases [145], further research studies are needed for obtaining “green” metallic nanoparticles and nanomaterials, using the natural extracts. In addition, for removing the actual drawbacks of drug-transporting vectors (such as phospholipids degradation, quick systemic exclusion, inadequate stability under prolonged storage, moderate efficiency for entrapping lipophilic compounds) [146], further in-depth studies are required. In addition to medical applications, phytosynthesized metallic nanoparticles could be applied as biosensors for the food industry or environment protection, offering on-site monitoring and providing real-time data, replacing the expensive equipment and sample processing, and providing the foundation for the development of next-generation catalysts or other important industrial applications.

## Figures and Tables

**Table 1:** Comparation of classical and modern extraction techniques for medicinal and aromatic plants.

Plant	Extraction Method	Extraction Conditions	Obtained Compounds	Extraction Yield	Reference
*Allium* *sativum* Linn.	Microwave-assisted hydro-distillation	Solvent: deionized water/diethyl ether 2:1; 100 g vegetal material; MP = 700 W; t = 30 min;	Diallyl sulfides (mono-, di-, tri-, and tetra-); Methyl allyl sulfides (di- and tri-); Vinyl dithiins	0.22%	[22]
	Ultrasound-assisted extraction	Solvent: diethyl ether (50 mL); F = 35 kHz; T = 25 °C; t = 30 min.		0.13%	
	Lickens–Nickerson apparatus	Solvent: water/diethyl ether = 1:10; 100 g. vegetal material; T = −10 °C; t = 2 h.		0.23%	
*Hippophae rhamnoides* L.	Solvent-free microwave-assisted extraction	400 g vegetal material atmospheric pressure; P = 400 W; T = 20–100 °C; t = 15 min.	Polyphenols with an increased yield of recovery for microwave extraction method	1147 mg GAE/g (d.w.)	[23]
	Classical extraction	Solvent: methanol 80% (50 mL); 5 g vegetal material; 8000 rpm; t = 5 min.		741.9 mg GAE/g (d.w.)	
*Matricaria chamomilla* L.	Subcritical water extraction	Solvent: water (300 mL); 10 g vegetal material; P = 30, 45 and 60 bars; T = 100 °C; t = 30 min;	Polyphenols	127–3226 mg/kg	[24]
	Maceration	Solvent: water (100 mL); 2.5 g vegetal material—oven-dried chamomile at low temperatures (i.e., 40 °C); T = 100 °C; t = 120 min.	Polyphenols	19.7 ± 0.5 mg/g (d.w.)	[25]
*Mentha* spp.	Microwave hydro- diffusion	Solvent-free; 500 g vegetal material; MP 1 W/g; F 2.45 GHz t = 20 min.	Essential oil	0.95%	[26]
	Soxhlet extraction	Solvent: water: ethanol = 3:7 (250 mL); 1.5 g dry plant material; T = 95 °C	Polyphenols	18,381–87,024 mg GAE/kg (d.w.)	[27]
*Origanum vulgare* L., 1753	Supercritical extraction	CO_2_ flow rate = 2.4 kg/h; 0.6 kg of vegetal material: CO_2_/plant ratio = 20 kg/kg; P = 30 MPa; T = 40 °C.	Carnosic acid	3.18 ± 0.40%	[28]
	Hydro-distillation	300 g vegetal material; t = 45 min	Essential oil rich in terpenes	0.75% (d.w.)	[29]
*Rosmarinus officinalis* L.	Maceration	Solvent: dichloromethane/ethanol = 3/1 (15 mL); 1 g vegetal material; T = 35 °C; t 3 h.	Carnosic acid, rosmarinic acid, carnosol	16.82; 0.12; 9.31 mg/g (f.w.)	[30]
	Supercritical fluid extraction	Solvent-free CO_2_ extraction; flow rate: 5 g/min; 100 g vegetal material; P = 100–300 bar; T = 40 °C; t = 3 h.	Carnosic acid, rosmarinic acid, camphor, 1,8-cineole	1.0730; 0.1242; 0.44; 0.029% (d.w.)	[31]
	Microwave-assisted extraction	Solvent: ethanol 96 %; 25 g milled leaves; Liquid/solid ratio = 6/1 (*v*/*w*); t = 7 min.	Carnosic acid, rosmarinic acid	3.3 ± 0.2 % (*w/v*) 3.1 ± 1.2 % (*w/v*)	[32]
*Salvia* *officinalis* L.	Supercritical CO_2_ extraction	Solvent-free CO2 extraction; flow rate: 1–3 kg/h; 50 g. of vegetal material; P = 15 or 20 MPa; T = 25 °C; t = 90 min.	Terpenes and phenolic compounds	0.659–5.477 % (*w/v*)	[33]
	Hydro distillation (Clevenger-type apparatus)	Solvent: water (1 L); 100 g vegetal material; t = 180 min.	Terpenes and phenolic compounds	2.0–2.1% (*v/w*)	[34]
	Maceration	Solvent: ethanol (70%)—25 mL; 5 g of vegetal material; t = 2 days;	Rosmarinic acid, carnosic acid, carnosol and methyl carnosate	n.p.	[35]
*Satureja hortensis* L.	Maceration	Solvent: ethanol 96% (300 mL); 10 g vegetal material; T = 22 °C; t = 7 days.	Phenolic compounds	125.34 mg GAE/g	[36]
	Soxhlet extraction	Solvent: ethanol 96% (600 mL); 75 g vegetal material; t = 8 h.		119.28 mg GAE/g	
	Microwave extraction	Solvent: ethanol 96% (100 mL); 5 g vegetal material; t = 30 min		147.21 mg GAE/g	
*Thymus* *daenensis* Celak. and *Thymus* *kotschyanus* Boiss. and Hohen	Hydro-distillation (Clevenger-type apparatus)	50 g vegetal material; t = 3 h.	Thymol, p-cymene, β-caryophyllene methyl carvacrol	1.2–2.4%	[37]
*Thymus munbyanus* Boiss. & Reut., 1852	Pressurized liquid extraction	Solvent: acetone; ethanol; water; 20 g. vegetal material; P = 45 MPa; T = 70 °C; t = 10 min	Oxygenated monoterpenoids; sesquiterpenoids and monoterpenoids	21.2 ± 0.6%	[38]

Where d.w.—dry weight; F—frequency; f.w.—fresh weight; GAE—gallic acid equivalents; MP—microwave power; n.p. —not provided by the authors; P—pressure; T—temperature; t—time.

**Table 2:** Some examples of the industrial applications of medicinal and aromatic plants (MAPs).

Plant	Bioactive Compounds	Presentation Form	Activity	Industrial Application	Reference
*Artemisia absinthium* L., *Calendula officinalis* L., *Lavandula vera* DC, *Syringa vulgaris* L.	Water-soluble vitamins	Emulsion	Antioxidant activity	Cosmetic industry	[117]
Cinnamon	Essential oil	Starch based edible film	Antibacterial activity (*Escherichia coli, Salmonella typhimurium* and *Staphylococcus aureus*)	Natural packaging	[128]
*Eryngium campestre* L.	Essential oil	Chitosan nanoparticle	-	Prolonging shelf life of sweet cherries	[107]
*Polylophium involucratum* (Pall.) Boiss.	Essential oil	Poly lactic acid/ nanochitosan composite film	Antimicrobial activity (*Pseudomonas* spp.)	Prolonging shelf life of chicken fillet	[109]
*Psiadia terebinthina* A.J. Scott	Essential oil	-	Melanin inhibition	Cosmetic industry	[120]
*Salvia miltiorrhiza* Bunge	Polysaccharide	-	Increase the number of leukocytes in blood	Increase growth performance of broilers	[129]
*Salvia rosmarinus* Spenn.	Aqueous extract	Whey protein concentrate/carboxymethyl cellulose/glycerol coatings	-	Coatings for sun flower seeds	[111]
*Satureja montana* L.	Essential oil	-	Protein oxidative stability Lipid oxidative stability	Prolonging shelf life of pre-cooked pork chops	[130]
*Thymus alternans* Klokov and *Teucrium montanum* subsp. *Jailae*	Essential oil	-	Insecticide (*Musca domestica* L., *Culex quinquefasciatus* Say and *Spodoptera littoralis* (Boisd.))	Natural insecticide	[113]
*Thymus capitatus* (L.) Hoffmanns et Link	Essential oil	Chitosan coatings	Antibacterial activity (*Aerobic mesophylls*, molds and yeasts)	Prolonged up to 1 day shelf life of strawberries stored under refrigeration conditions (5 ± 0.5 °C)	[105]
*Thymus capitatus* (L.) Hoffmanns et Link	Essential oil	Nano-emulsion	Antibacterial activity (*Staphylococcus aureus*)	Food preservative	[106]
*Thymus kotschyanus* Boiss. & Hohen.	Essential oil	Chitosan–starch composite film	Antibacterial activity (*Pseudomonas* spp. and *Listeria monocytogenes*)	Prolonging shelf life of beef during storage on a period of 21 days at 4 °C	[108]
*Thymus serpyllum* L.	Essential oil	-	Antimicrobial activity (*Escherichia coli, Salmonella typhimurium, Staphylococcus aureus* and *Pseudomonas aeruginosa*)	Ground pork patty	[131]
-	Limonene, linalool, menthol, and thymol	Chitosan nanoparticles	Antimicrobial activity (*Escherichia coli* and *Salmonella typhimurium*)	Preservation of minced meat	[132]

## Data Availability

No new data were created or analyzed in this study. Data sharing is not applicable to this article.
